# Unraveling the Early Events of Amyloid-β Protein (Aβ) Aggregation: Techniques for the Determination of Aβ Aggregate Size

**DOI:** 10.3390/ijms13033038

**Published:** 2012-03-07

**Authors:** N. Elizabeth Pryor, Melissa A. Moss, Christa N. Hestekin

**Affiliations:** 1 Ralph E. Martin Department of Chemical Engineering, 3202 Bell Engineering Center, University of Arkansas, Fayetteville, AR 72701, USA; E-Mail: npryor@uark.edu; 2 Department of Chemical Engineering, 2C02 Swearingen Engineering Center, University of South Carolina, Columbia, SC 29208, USA; E-Mail: MOSSME@cec.sc.edu

**Keywords:** amyloid, capillary electrophoresis, centrifugation, fluorescence correlation spectroscopy, light scattering, mass spectrometry, polyacrylamide gel electrophoresis, oligomer, size exclusion chromatography, Western blotting

## Abstract

The aggregation of proteins into insoluble amyloid fibrils coincides with the onset of numerous diseases. An array of techniques is available to study the different stages of the amyloid aggregation process. Recently, emphasis has been placed upon the analysis of oligomeric amyloid species, which have been hypothesized to play a key role in disease progression. This paper reviews techniques utilized to study aggregation of the amyloid-β protein (Aβ) associated with Alzheimer’s disease. In particular, the review focuses on techniques that provide information about the size or quantity of oligomeric Aβ species formed during the early stages of aggregation, including native-PAGE, SDS-PAGE, Western blotting, capillary electrophoresis, mass spectrometry, fluorescence correlation spectroscopy, light scattering, size exclusion chromatography, centrifugation, enzyme-linked immunosorbent assay, and dot blotting.

## 1. Introduction

Protein aggregation leads to the formation of insoluble fibrous aggregates, termed amyloids, which are commonly associated with disease. However, understanding of the mechanism by which proteins aggregate has remained elusive. Although larger aggregates, including fibrils, remain important for clinical determination [1,2], small oligomeric aggregates are of interest due to their potentially toxic nature and hypothesized role in disease progression. However, the study of oligomers is complex due to the fact that these early aggregates are highly unstable, present at low concentrations, and difficult to isolate.

Among the diseases to which amyloids contribute are Alzheimer’s disease (AD), Parkinson’s disease, prion diseases, Type II diabetes mellitus, Huntington’s disease, as well as many others [3]. The clinical presentation of each amyloid disease is very different, yet the presence of amyloid fibrils is a common characteristic of each disease. These amyloid fibrils exhibit a cross β-sheet structure in which the β-strands are oriented perpendicular to and hydrogen bonding is oriented parallel to the long axis of the fibril [4–9]. In addition, it has been shown that the amyloidogenic proteins amyloid-β (Aβ), α-synuclein, huntingtin, prion, and islet amyloid polypeptide (IAPP) form structurally similar soluble oligomeric species, which share an epitope recognized by oligomer-specific antibodies [10,11]. The commonalities shared by each amyloid disease protein suggest that studying the aggregation of one amyloid protein could provide insight into the general aggregation mechanism of other amyloid proteins.

**Figure 1:**
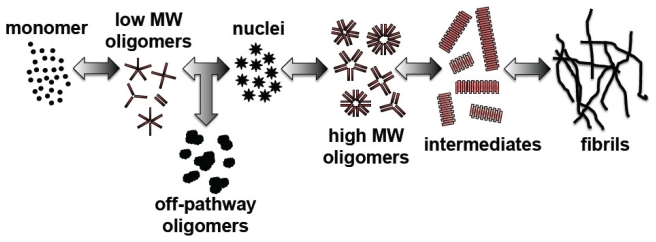
The Aβ aggregation process. Aβ monomer self-assembles into low molecular weight oligomeric species that can give rise to either off-pathway oligomers or nuclei of an undetermined size. Nuclei, which arise within the rate-limiting step of the Aβ aggregation pathway, will increase in size to form high molecular weight oligomers, soluble aggregation intermediates, and finally the fibrillar aggregates that deposit in AD brain to yield amyloid plaques.

AD is the most common cause of dementia and the most prevalent neurodegenerative disorder [12,13]. The neurodegenerative effects of AD are hypothesized to arise from Aβ, a partially folded protein that aggregates during the disease process. Aβ was first identified by Masters *et al.* as the aggregated protein [14] deposited within plaque cores found in AD brain. In its monomeric form, this protein may be harmless [15]. However, Aβ monomer can self-assemble via a nucleation-dependent pathway into Aβ oligomers, larger Aβ aggregation intermediates, and eventually the fibrillar aggregates that deposit in the brain (Figure 1) [5,16–18]. Steps within the Aβ aggregation pathway are reversible, such that deposited fibrils could give rise to soluble oligomers and intermediates. Soluble aggregate species that appear between monomer and insoluble fibrils have been termed within the literature as oligomers [19], micelles [20], amyloid-derived diffusible ligands (ADDLs) [21,22], βamy balls [23], amylospheroids (ASPDs) [24], and protofibrils [25,26], and the aggregate sizes associated with these definitions overlap in range. Smaller species are most commonly referred to as oligomers, including both low molecular weight and high molecular weight species, while larger intermediates are often referred to as protofibrils. Controversy exists concerning the exact size of the nucleus formed within the rate-limiting step of the aggregation pathway; however, most reports speculate that the nucleus is oligomeric in nature [27–29]. In addition to oligomers formed along the aggregation pathway, off pathway oligomers and higher order assemblies, which fail to give rise to an organized fibril structure, have also been identified [29,30].

Aβ proteins comprised of either 40 or 42 amino acids, termed Aβ_1-40_ and Aβ_1-42_, are the major components found in amyloid plaques [31]. Aβ_1-42_ has implications for the formation of initial aggregates, while Aβ_1-40_ is more soluble and is the main circulating form in normal plasma and cerebrospinal fluid (CSF) [32]. Controversy currently exists over the direct effect Aβ has on neurodegeneration, but it is theorized that soluble aggregates of Aβ, rather than monomers or insoluble fibrils, may be responsible for the cellular pathology associated with AD [33–35]. This hypothesis is supported by experimental observations *in vitro* which show that soluble aggregates formed by synthetic Aβ_1-40_ and Aβ_1-42_ can induce cellular dysfunction and toxicity in cultured cells [21,36,37] and *in vivo* where Aβ dodecamers (Aβ*56) have been isolated from the brains of transgenic mice and shown to induce memory deficits [38]. In addition, soluble Aβ aggregates generated in cell culture drastically inhibit hippocampal long term potentiation in rats [39]. Furthermore, data from mouse models show a poor correlation between the levels of insoluble Aβ fibrils and disease severity [40]. It is now more widely accepted that soluble Aβ oligomers impair cognitive function and, in addition to synapse loss, correlate most accurately with the stage of neurological impairment [11,41–43] However, the progression from monomer to oligomer to insoluble Aβ aggregates is not well understood. Therefore, it is important to develop an analytical tool that is suitable for analysis of the Aβ aggregation process.

A range of techniques are available to study the different stages of the Aβ aggregation process. These techniques fall into three main categories: (1) Methods for the quantitative detection of monomeric and oligomeric Aβ sizes; (2) Methods for the qualitative detection and characterization of oligomeric Aβ species; (3) Methods for the qualitative detection of Aβ fibrils. As a result of the imminent need to understand oligomerization events, the focus of this review is on techniques from the first and second categories, which give information about Aβ oligomeric species formed during aggregation. Accumulating evidence suggests that these Aβoligomeric species play a role in AD progression and severity. Therefore, it is important to gain a better understanding of the formation of smaller Aβ species in order to halt the progression of AD. The ability to identify and quantify the size of these Aβ oligomeric species without disrupting their structure is of utmost importance in order to effectively study the aggregation process and develop treatments that target these pivotal oligomerization events. Accordingly, this review focuses primarily upon techniques that have been employed in the study of *in vitro* aggregation of Aβ. Currently, a commonly used technique for the quantification of Aβ oligomer sizes within *in vitro* studies is polyacrylamide gel electrophoresis (PAGE). Other techniques that have been applied for determining the size of Aβ oligomers include Western blotting, capillary electrophoresis, mass spectrometry, fluorescence correlation spectroscopy, light scattering, centrifugation, and size exclusion chromatography (SEC). Furthermore, techniques including enzyme-linked immunosorbent assay (ELISA) and dot blot have been applied to identify Aβ oligomers, but give no size estimates. In the subsequent sections, we will discuss the application of each of these techniques to study Aβ oligomers.

## 2. Electrophoretic Techniques for the Quantification of Aβ Oligomer Sizes

### 2.1. SDS- and Native-PAGE

SDS-PAGE is the most common electrophoretic technique used for Aβ oligomer size determination in protein aggregation studies. Furthermore, a review by Bitan *et al.* cited SDS-PAGE as the most common method used to characterize toxic protein oligomers [44]. SDS-PAGE relies on the ability of SDS, a negatively charged detergent, to bind to the protein of interest. This binding typically results in the removal of secondary, tertiary, and quaternary structures from the protein. The SDS groups attach to the protein in a nearly uniform manner that gives the protein a charge approximately proportional to its length, thereby allowing for size based separations. Following the gel electrophoretic separation of proteins, the gel may be stained with a dye such as Coomassie Brilliant Blue or silver stain.

**Figure 2:**
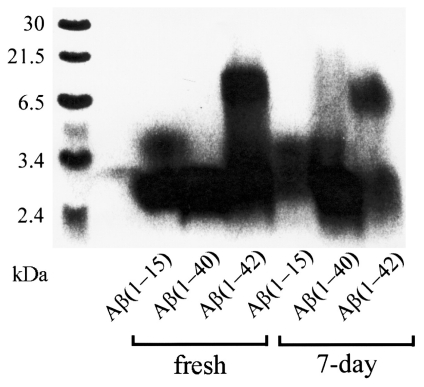
Tricine-SDS-PAGE analysis of the aggregation states of Aβ peptides freshly dissolved or incubated for 7 days. Aβ_1-42_ exhibits bands at 5–20 kDa in both freshly prepared samples and samples incubated for 7 days. Aβ_1-40_ incubated for 7 days also exhibits a smear at higher molecular weights, which is absent in freshly prepared samples. Reprinted from [46], with permission from Elsevier.

Many research groups have utilized SDS-PAGE, as a standalone technique, to study the evolution of Aβ species over time. A study by Ying *et al.* used SDS-PAGE to separate oligomers formed by 100 μM Aβ_1-42_ incubated at 4 °C for 1 day [45]. SDS-PAGE revealed bands for monomer (4.5 kDa), trimer/tetramer (16.5 kDa), and higher molecular weight intermediates (>83 kDa) that appeared as a smear. The oligomer pattern of freshly dissolved Aβ peptides and Aβ peptides after a 7 day incubation have been observed by Satoh *et al.* [46]. Both Aβ_1-40_ and Aβ_1-42_ peptides incubated for 7 days as well as the freshly dissolved Aβ_1-42_ peptide exhibited a range of species from 5–20 kDa (Figure 2). However, the resolution of these species was low due to gel smearing. Smearing in these gels may be due to the resolution limitations of the gel or could be due to continuous associations and disassociations of the aggregating species occurring during the electrophoresis analysis. Whatever the cause, gel smearing interferes with the ability to identify a particular species and is often overcome by combining SDS-PAGE with another technique (see Sections 2.2 and 2.3).

Although the anionic micelles formed by SDS enhance separation, they can also induce non-native behavior. SDS has been reported to accelerate the generation of Aβ fibrils. Sureshbabu *et al*. have shown that Aβ_1-42_ freshly prepared in phosphate buffered saline exhibits monomer, trimer (~13.5 kDa), and tetramer (~18 kDa) bands when analyzed via Western blotting [47]. The addition of 1.5 mM SDS to the sample produced bands at 20 and 50 kDa. They proposed that the addition of 1.5 mM SDS causes Aβ_1-42_ to develop a partial helical structure whose hydrophobicity induces aggregation. One way to counter this phenomenon is to add urea to the sample to further denature the peptide and prevent aggregation. However, the migration behavior of Aβ peptides in urea SDS-PAGE is inconsistent. A study by Kawooya *et al*. showed that the Aβ peptide exhibits an unusual electrophoretic mobility in urea SDS-PAGE that is proportional to the sum of the hydrophobicity consensus of the peptide rather than the number of amino acids in the peptide [31]. Therefore, under these conditions SDS-PAGE may provide information about the hydrophobicity of the peptide and not the size. The drawbacks of SDS-PAGE may be overcome by using native-PAGE to separate various Aβ sizes under conditions that allow the protein to remain in a native state.

Native or “non-denaturing” gel electrophoresis is similar to SDS gel electrophoresis, except this technique is run in the absence of SDS. With native-PAGE, protein mobility depends on both charge and hydrodynamic size. This differs from SDS-PAGE, where protein mobility depends primarily on molecular mass. Since Aβ aggregation is a process that involves changes in protein conformation, native-PAGE is often a suitable technique to detect various sizes of Aβ species. A study by Iurascu *et al.* used both SDS-PAGE and Tris-tricine PAGE to analyze the species formed by a solution of Aβ_1-40_ solubilized in fibril growth buffer at pH 7.5 for 5 days at 37 °C [48]. They found that SDS-PAGE was able to detect Aβ_1-40_ monomeric species, Aβ_1-40_ oligomeric species of 20 kDa, and high molecular weight aggregates >98 kDa. In contrast, Tris-tricine PAGE was able to separate these Aβ oligomers into monomer, dimer, trimer, and high molecular weight Aβ sizes. Klug *et al.* have also compared native and SDS-PAGE analyses of Aβ aggregation [49]. They observed the presence of oligomers and high molecular weight species using native-PAGE with the majority of Aβ species observed in the high molecular mass region of the gel. In contrast, SDS-PAGE showed lower molecular weight species (<14 kDa) with only trace amounts of high molecular weight species (>50 kDa), suggesting that the removal of higher order protein structures by SDS may destabilize aggregates. The differences between native-PAGE and SDS-PAGE highlight the importance of examining more than one method for studies of the various Aβ aggregate sizes formed throughout the aggregation process.

### 2.2. SDS-PAGE in Combination with Western Blotting

Western blotting is a popular technique used to further process samples after electrophoretic separation. This technique provides a more sensitive detection of separated proteins. This detection is achieved by transferring separated proteins to a membrane where they are detected using antibodies specific to the protein of interest. Antibodies may be either monoclonal or polyclonal and are typically specific for a particular part of the Aβ sequence or a particular amyloid conformation. Some common antibodies and their recognition motifs are listed in Table 1. Selecting the proper antibody is an important consideration in order to achieve detection of the desired Aβ species or aggregation state.

Numerous research groups have utilized Western blot analyses of SDS-PAGE separations to characterize SDS-stable Aβ assemblies [21,39,45,50–53,55]. Ryan *et al*. analyzed Aβ_1-42_ oligomer preps via silver staining and immunoblot with the 6E10 antibody [52]. The band intensity for monomer, trimer, and tetramer bands was similar for both methods. However, 46 and 56 kDa intermediate sized oligomers were more apparent in the immunoblot analysis. Moore *et al*. have also found that immunoblot stains of Aβ_1-42_ oligomers yield better results than silver stains [54].

SDS-PAGE with Western blotting has also been used to monitor the formation of Aβ oligomers in cell culture. A study by Walsh *et al*. employed SDS-PAGE followed by Western blotting to probe the formation of Aβ oligomers in APP-expressing Chinese hamster ovary (CHO) cells [50]. Bands corresponding to ~4, 6, 8, and 12 kDa were obtained using the monoclonal antibody 6E10. However, it was necessary to concentrate the Aβ protein via immunoprecipitation with an Aβ-specific antibody prior to performing electrophoretic separation.

Within *in vitro* studies of Aβ aggregation, Aβ is typically solubilized in 1,1,1,3,3,3-hexafluoro-2-propanol (HFIP) to break up any residual aggregates that may be present in solution [61]. The HFIP is allowed to evaporate, and the peptide film is either resuspended in an organic solvent such as dimethyl sulfoxide (DMSO) and diluted into culture media or resuspended in a buffer solution such as phosphate buffered saline (PBS). Following incubation, samples are analyzed to detect the presence of oligomeric species. Dahlgren *et al*. utilized such an Aβ_1-40_ oligomer preparation employing DMSO and F12 culture media with incubation at 4 °C for 24 h [53]. Western blot analysis using the 6E10 antibody showed bands corresponding to monomer and tetramer. Similar results were obtained by Stine *et al*. using the same sample preparation [51]. Walsh *et al*. utilized an Aβ_1-40_ oligomer preparation in PBS (pH 7.4) at 37 °C [55]. After 5 days, Western blot analysis using the antibody 2G3 showed bands corresponding to monomer, dimer, and tetramer. However, intermediate sizes of oligomeric species >20 kDa were not obtained.

**Figure 3:**
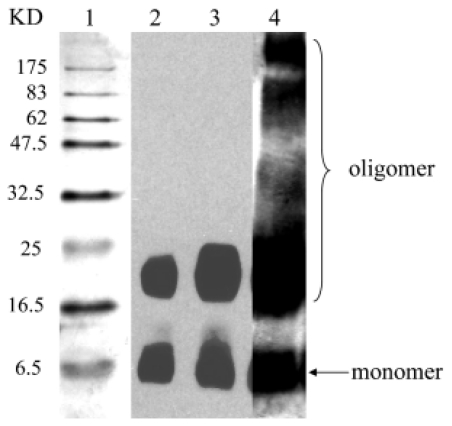
Aβ_1-42_ oligomers obtained upon incubation at 4 °C for 24 h. A 5 mM Aβ_1-42_ sample was prepared in DMSO and diluted to 100 μM in Ham’s F12 medium without phenol red. Oligomer mixture was separated by 15% SDS-PAGE, transferred to nitrocellulose membranes, and probed with monoclonal antibody A8 (Lanes 2 and 3) or 6E10 (Lane 4). Sample in Lane 2 was heat denatured prior to analysis, while sample in Lane 3 was untreated. Reprinted from [45] with permission. The publisher for this copyrighted material is Mary Ann Liebert, Inc. publishers.

In addition to Aβ_1-40_, oligomeric Aβ_1-42_ species formed *in vitro* have been well characterized using Western blot analyses. Stine *et al*. studied the formation of Aβ_1-42_ oligomers using two different antibodies, 6E10 and 4G8 [51]. At 0 h, bands for monomer, trimer, and a faint tetramer band were obtained. After 24 h, these bands were more intense and a smear corresponding to oligomeric species ranging from 30 to 70 kDa was present. Furthermore, no differences in the band patterns obtained using the 6E10 and 4G8 antibodies were observed. Dahlgren *et al*. obtained comparable 24 h incubation results using the same oligomer preparation as Stine *et al.* [53]. In addition, similar 0 and 24 h results were obtained by Ryan *et al*. using a monomer preparation with dilution into PBS and an oligomer preparation with dilution into cold PBS + 0.05% SDS [52]. Stine *et al*. also examined the effect of temperature and ionic strength on the oligomeric band pattern obtained after incubation of 100 μM Aβ_1-42_ for 24 h. An increase in temperature from 4 to 37 °C resulted in a decreased intensity of monomer and trimer bands and an increased intensity of the tetramer band. In addition, a smear for oligomeric species ranging from 30 to 70 kDa appeared at 25 °C with increased intensity at 37 °C. The effect of ionic strength was probed using the oligomer preparation at 37 °C with incubation for 24 h in either 10 mM Tris (pH 7.4) or 10 mM Tris supplemented with 150 mM NaCl. Both preparations yielded bands for monomer, trimer, and tetramer. However, the oligomer preparation in 10 mM Tris gave an intense oligomer smear from 30 to 97 kDa while the preparation in 10 mM Tris supplemented with 150 mM NaCl showed a less intense oligomer smear from 40 to 50 kDa. Ying *et al*. have also utilized the same Aβ_1-42_ oligomer preparation as Stine *et al*. but employed for detection the monoclonal antibody A8, which is specific for oligomers [45]. A smear for oligomeric species ranging from 16.5 to 25 kDa was observed with antibody A8 (Figure 3, lanes 2 and 3). A poorer resolution of oligomers and larger species were obtained using the 6E10 antibody (Figure 3, lane 4). These results show that 6E10 may be reacting more strongly with higher molecular weight oligomers or that these antibodies bind preferentially to different sizes of Aβ_1-42_ oligomers. While Western blotting does facilitate detection of intermediate Aβ oligomers, the presence of a gel smear in many of the studies outlined above indicates that this technique does not allow quantification of individual sizes of oligomers in this range.

### 2.3. SDS-PAGE in Combination with Other Techniques

SDS-PAGE has been used in combination with oligomer stabilization techniques. One such technique that has been applied by Bitan *et al*. is Photoinduced Cross-Linking of Unmodified Proteins (PICUP) [62]. PICUP was developed in the Kodadek laboratory in 1999 to study proteins that naturally form stable homo- or heterooligomers [63]. This technique provides a snapshot of different oligomer species present in solution at different times. Protein cross-linking is achieved via the visible light excitation of a tris(2,2′-bipyridyl)dichlororuthenium(II) complex which, through a series of steps, leads to the generation of a free protein radical [62,64]. This radical can attack an unmodified neighboring protein and form a covalent bond. Therefore, PICUP can be used to covalently freeze components of the sample, and these components may be separated and analyzed via techniques such as SDS-PAGE [62].

**Figure 4:**
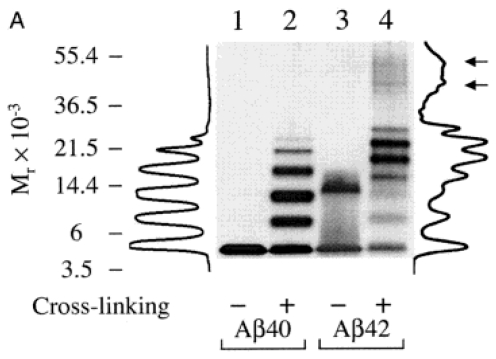
SDS-PAGE analysis of non-cross-linked (lanes 1 and 3) and cross-linked (lanes 2 and 4) Aβ_1-40_ and Aβ_1-42_. Densitometric intensity profiles of lanes 2 and 4 are shown on the right and left sides of the gel, respectively. Molecular weight standards are shown on the left in kDa. Adapted from [62] with permission. Copyright (2003) National Academy of Sciences, U.S.A.

Bitan *et al*. have applied PICUP to compare low molecular weight fractions of Aβ_1-40_ and Aβ_1-42_, where these fractions were isolated by SEC and analyzed via SDS-PAGE [65]. Aβ_1-40_ exhibited bands for monomer, dimer, trimer, and tetramer with more faint bands for pentamer and heptamer (Figure 4, lane 2). A distinctly different low molecular weight Aβ_1-42_ oligomer size distribution, consisting of three groups of oligomers of varying band intensity, was obtained (Figure 4, lane 4). This pattern led to the conclusion that the initial phase of Aβ_1-42_ oligomerization involves the formation of pentamer/hexamer subunits which then associate to form larger oligomers and intermediates, or protofibrils [65]. Furthermore, they found that for Aβ_1-40_, monomer through tetramer were preexisting species in solution, while pentamer through heptamer were formed via a diffusion-dependent reaction of these preexisting species with free monomer. Their results verified that PICUP was capable of “freezing” preexisting oligomers but was also capturing oligomeric species which were not formed under typical aggregation conditions, thereby misrepresenting the true Aβ_1-40_ oligomerization pattern. In addition, this study examined samples that were not cross-linked via PICUP before separation by SDS-PAGE. A single monomer band was obtained for Aβ_1-40_ (Figure 4, lane 1), while Aβ_1-42_ exhibited only bands for monomer and trimer (Figure 4, lane 3). These results indicate that oligomers not stabilized via PICUP were underestimated by SDS-PAGE results.

SDS-PAGE has also been combined with SEC to investigate Aβ aggregation [25,66,67]. A study by Podlisny *et al*. used SDS-PAGE and SEC to observe the aggregation process of Aβ_1-40_ secreted from CHO cells [66]. Soluble, SDS-stable aggregates of 6–25 kDa, were detected during the first 4.5 h of incubation at 37 °C via added radioiodinated synthetic Aβ_1-40_ at low nanomolar concentrations. These 6–25 kDa Aβ oligomers represented ~18% of the total Aβ signal via SDS-PAGE and ~31% of the total Aβ signal via SEC. This low conservation of the Aβ gel signal over time to oligomeric species again indicates that SDS-PAGE underestimates the amount of aggregation. A study by Walsh *et al.* compared size estimations via SEC to those obtained by analyzing these SEC fractions by SDS-PAGE [25]. Aβ_1-40_ and Aβ_1-42_ were dissolved in Tris-HCl (pH 7.4) and incubated for 48 and 6 h, respectively, at room temperature. SEC fractions corresponding to Aβ_1-40_ dimers, protofibrils, and fibrils produced a single band at ~4 kDa on SDS-PAGE. The SEC fraction for Aβ_1-42_ dimers produced a single SDS-PAGE band at ~4 kDa, while the SEC fraction for Aβ_1-42_ protofibrils and fibrils produced a ladder of sizes ranging only from monomer to pentamer. These results suggest that SDS-PAGE may not accurately detect Aβ aggregate sizes produced throughout aggregation.

### 2.4. Summary of SDS-Based Methods

As a standalone technique, SDS-PAGE is able to detect Aβ_1-42_ species ranging from monomer to tetramer. Native-PAGE has been used to separate Aβ_1-40_ species ranging from monomer to pentamer. However, for higher order oligomers, these techniques only give a range of sizes that appear as a smear on the gel. SDS-PAGE is often coupled with other techniques such as Western blotting and PICUP to enhance the resolution of Aβ sizes. By coupling SDS-PAGE to these techniques, a better resolution of Aβ_1-40_ species which appear as individual gel bands corresponding to monomer, dimer, trimer, and tetramer and Aβ_1-42_ species which appear as individual gel bands corresponding to monomer, trimer, tetramer, and hexamer has been obtained. However, the resolution of intermediate sized Aβ oligomers ranging from 30–70 kDa by PAGE remains a significant challenge. The addition of SDS may also lead to complications including the acceleration of aggregation and the increased instability of oligomers, thereby misrepresenting the distribution of Aβ oligomeric species.

### 2.5. Capillary and Microfluidic Capillary Electrophoresis

Capillary electrophoresis (CE) is another electrophoretic technique employed for size based separations of Aβ. CE offers a fast and highly efficient separation of molecules with a broad range of properties thereby making it well suited for the separation of different sizes of protein aggregates [68]. CE separates molecules based on electrophoretic mobility, which results from differences in charge, shape, and/or size, and may be used either with or without SDS. Thus, CE allows a highly efficient separation and resolution of native forms of Aβ species, thereby overcoming the problem of gel smearing in many SDS and native-PAGE gel separations. CE detection typically uses either ultraviolet (UV) absorbance or laser induced fluorescence (LIF) to detect proteins. UV can detect proteins without any additional labeling, but typically has a lower sensitivity than LIF. LIF usually requires labeling of the molecules, but is highly sensitive, with previous reports of CE-LIF detection of double-stranded DNA down to the pg/μL range [69,70]. The ability to detect biomolecules at these low concentrations is necessary for the analysis of physiologically relevant protein concentrations.

**Figure 5:**
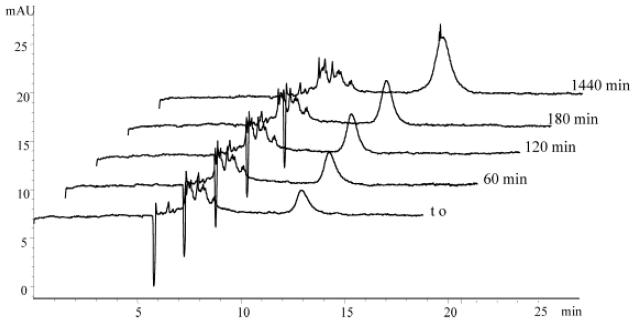
Electropherograms for Aβ_1-42_ species formed in room temperature PBS (pH 7.4) at different elapsed aggregation times from *t*_0_. CE was performed with 50 mbar pressure injection for 8 s with separation at 16 kV. Molecular weights corresponding to each peak were determined using Microcon centrifugal filter units with molecular weight cutoffs of 3, 10, 30, and 50 kDa. Peaks with migration times of 5–10 min represent monomers to undecamers/dodecamers (3–50 kDa) and peaks with migration times of 10–15 min represent larger aggregates (>50 kDa). Reprinted from [72] published by John Wiley and Sons, © 2004 WILEY-VCH Verlag GmbH & Co. KGaA.

CE with UV detection has been utilized by various researchers to detect Aβ species from monomers to large aggregates. Verpillot *et al*. used CE-UV to separate monomeric Aβ ranging in size from 37–42 residues and differing in length by a single residue, however they did not examine Aβ aggregation [71]. A study by Sabella *et al.* applied CE-UV with an SDS rinse for the detection of Aβ_1-40_ and Aβ_1-42_ oligomers formed in PBS (pH 7.4) at room temperature [72]. At 0 h, peaks for Aβ_1-42_ oligomers in a size range from monomers to undecamers (~50 kDa)/dodecamers (~54 kDa) and larger aggregates were obtained (Figure 5, *t*_0_). A similar peak pattern was obtained over an incubation time period of 24 h with an increase in intensity of the higher molecular mass (>50 kDa) oligomer peak (Figure 5, *t* = 1440 min). However, resolution of individual species, especially in the larger aggregate peak, was not achieved. Compared to Aβ_1-42_, the peaks for Aβ_1-40_ were better resolved, but a drastically different peak pattern was observed. At 0 h, three peaks ranging in size from 3 to 30 kDa were obtained. A decrease in the intensity of the 10 to 30 kDa peak was observed over an incubation period of 24 h with the disappearance of all peaks after 48 h. This result shows that CE-UV is capable of detecting small Aβ_1-40_ species and intermediate oligomeric Aβ_1-42_ species. In addition, the CE electrophoretic profiles of Aβ_1-40_ and Aβ_1-42_ differ significantly, supporting observations by PAGE that these two proteins differ in their early stages of aggregation.

Picou *et al.* also observed substantial differences in the CE-UV electrophoretic profiles of Aβ_1-40_ and Aβ_1-42_ [73]. Two different preparations typically employed to form Aβ monomer or fibril were used. The Aβ_1-40_ monomer preparation yielded a single monomer peak with a molecular weight of 4.3 kDa. In contrast to Aβ_1-40_, the Aβ_1-42_ monomer preparation gave peaks for both monomer and fibrillar species. A peak pattern similar to the Aβ_1-42_ monomer preparation was also obtained for the Aβ_1-40_ fibril preparation. The Aβ_1-42_ fibril preparation produced multiple aggregate peaks and no monomer peak. Although this study was able to separate Aβ monomer from mature fibrils, the detection of oligomeric Aβ species was not achieved.

LIF detection has also been utilized as a more sensitive means of identifying lower concentrations of Aβ aggregate species separated using CE. A study of the aggregation patterns of Aβ_1-42_ using CE-LIF was conducted by Kato *et al.* [74]. The fluorescent dye thioflavin T (ThT) was used to detect two different Aβ_1-42_ aggregate sizes with a 5 min analysis time [74]. In addition, this study examined the effect of seeding a freshly prepared Aβ_1-42_ sample with a fibrillar Aβ_1-42_ seed. For samples without a seed, a broad peak was observed with CE-LIF as opposed to seeded samples that contained both a sharp and broad peak, although no specific sizes were determined.

In addition to CE-LIF, microfluidic capillary electrophoresis (MCE) has been used to study Aβ. MCE is similar to CE except operates on a much smaller scale. The advantages of MCE over conventional electrophoresis methods include low sample consumption and a strong potential for automation and integration [75,76]. MCE has been utilized to study Aβ monomeric species. A study by Mohamadi *et al.* utilized MCE-LIF for the separation of five Aβ isoforms (Aβ_1-37_, Aβ_1-38_, Aβ_1-39_, Aβ_1-40_, and Aβ_1-42_) [77]. However, MCE has yet to be applied for the study of Aβ oligomers.

CE as a technique for the detection of Aβ species formed throughout aggregation is still in its early stages. CE-UV has been utilized to detect small Aβ_1-40_ species ranging from 3–30 kDa as well as to separate Aβ_1-40_ monomer from fibrillar species. Aβ_1-42_ species ranging from 3–50 kDa and >50 kDa have been detected using CE-UV. In addition, the separation of Aβ_1-42_ monomer from fibrillar species has been achieved using CE-UV, and the separation of two different Aβ_1-42_ fibrils has been accomplished with CE-LIF. The development of MCE has prompted researchers to apply this technique to the study of Aβ, with initial investigations demonstrating the separation of five Aβ isoforms differing in length by a single residue. The ability of CE to detect sizes from monomers to fibrils offers the potential to monitor the amyloid aggregation process over time, and the use of LIF provides the potential for examining physiologically relevant concentrations. However, further improvements to this technique must be made in order to enhance the resolution of intermediate sized Aβ species.

## 3. Spectroscopic Techniques for the Quantification of Aβ Oligomer Sizes

### 3.1. Mass Spectrometry

Mass spectrometry (MS) is a widely used technique for the detection of monomeric and oligomeric Aβ. In MS, the sample undergoes vaporization, and components are ionized by impacting them with an electron beam. Ions are separated by their mass-to-charge ratio using electromagnetic fields, and the ion signal is processed into a mass spectrum characteristic of the analyte. MS uses a variety of ionization sources depending on the sample state. For vapor samples, the most common source used to generate gas-phase ions is a radioactive ionization (RI) source [78,79]. However, other ion sources such as corona discharge ionization (CDI) [80,81], photoionization (PI) [80,82], and secondary electrospray ionization (SESI) [83–86] have been used as well. The most commonly used ionization source for liquid samples is electrospray ionization (ESI) [83–86], and for solid samples matrix assisted laser desorption ionization (MALDI) [87–90] and laser desorption ionization (LDI) [91–93] are widely used ionization sources. In addition, there are various types of mass analyzers that process the ion signal into a mass spectrum. These include time-of-flight, quadrupole, ion trap, Fourier transform ion cyclotron, magnetic sector, and tandem instruments as recently reviewed by Kanu *et al.* [94]. The most common MS techniques used for protein analyses are MALDI-MS and ESI-MS.

### 3.2 Matrix-Assisted Laser-Desorption Ionization (MALDI)-MS

MALDI-MS may be combined with other separation techniques such as SDS-PAGE to provide more quantitative size estimates. Iurascu *et al*. utilized SDS-PAGE in combination with MALDI-MS to analyze a solution of Aβ_1-40_ solubilized in fibril growth buffer at pH 7.5 for 5 days at 37 °C [48]. MALDI-MS indicated that the soluble fraction contained two different ion mobilities, indicative of oligomerization. Parallel analysis using SDS-PAGE and Tris-tricine PAGE revealed the presence of oligomeric Aβ_1-40_ of ~20 kDa (pentamer). A study by Maji *et al*. subjected wild-type and tyrosine substituted Aβ_1-40_ and Aβ_1-42_ to PICUP and quantified the resulting aggregate sizes via MALDI-MS and SDS-PAGE [95]. SDS-PAGE yielded wild-type Aβ_1-40_ bands for monomer through hexamer. However, MALDI-MS was only able to attain masses for the monomer through tetramer bands, while masses for the pentamer and hexamer bands could not be measured. This inconsistency could be attributed to the presence of very small quantities of pentamer and hexamer. Alternatively, these species may not be desorbed from the MALDI matrix as readily as smaller oligomers. In addition, MALDI-MS spectra of tyrosine substituted Aβ_1-42_ oligomers were not obtained, suggesting that either these oligomers could not be incorporated into the MALDI matrix due to their exceptional hydrophobicity or their covalent or weak noncovalent interactions were disrupted by the desorption/ionization process. These results show that although MALDI-MS may be used to quantify Aβ oligomers, this technique does have drawbacks including limited matrix interactions as well as the inability to distinguish molecules with overlapping charge-to-mass ratios, expense, and labor intensive analyses [96,97]. In addition, since MALDI is typically coupled with a pre-separation step such as SDS-PAGE, its detection capabilities may vary depending on the pre-separation technique used.

### 3.3. Electrospray Ionization (ESI)-MS

**Figure 6:**
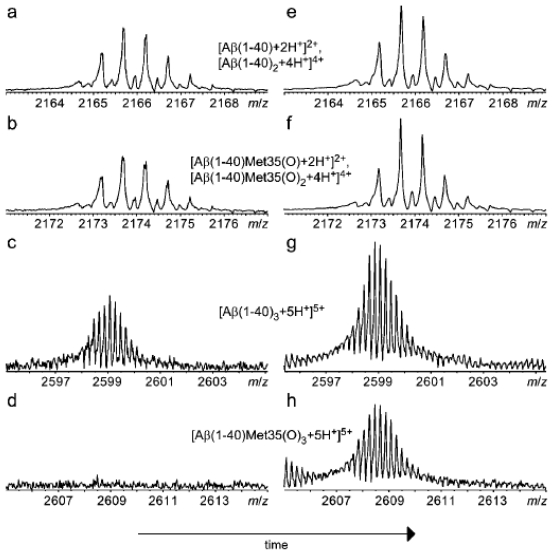
ESI-Mass spectra of 4.0 μM freshly dissolved Aβ_1-40_ (a and c), freshly dissolved Aβ_1-40_Met35(O) (b and d), Aβ_1-40_ and Aβ_1-40_Met35(O) incubated for 41 min (e and f), and Aβ_1-40_ and Aβ_1-40_Met35(O) incubated for >95 min (g and h). Aβ_1-40_ samples were dissolved in H_2_O and Aβ_1-40_Met35(O) samples were dissolved in H_2_O and 2.7% H_2_O_2_. Reprinted with permission from [98]. Copyright (2002) The American Society for Biochemistry and Molecular Biology.

ESI-MS has been used to analyze liquid Aβ samples. Palmblad *et al*. have utilized ESI-MS to study the effect of Met-35 oxidation on the formation of Aβ_1-40_ oligomers [98]. They found that freshly dissolved Aβ_1-40_ and Aβ_1-40_Met35(O) both exhibited monomers and dimers (Figure 6, panels a and b). In addition, Aβ_1-40_ and Aβ_1-40_Met35(O) incubated for 41 min exhibited similar monomer and dimer signals (Figure 6, panels e and f). In contrast, trimers and tetramers were detected for freshly dissolved Aβ_1-40_ (Figure 6, panel c) whereas these species were not detectable for freshly dissolved Aβ_1-40_Met35(O) (Figure 6, panel d). However, after >95 min of incubation, Aβ_1-40_ and Aβ_1-40_Met35(O) exhibited similar trimer and tetramer signals (Figure 6, panels g and h). These results suggest that Met-35 oxidation slows a conformational change that may be necessary for early formation of Aβ_1-40_ trimers. Although ESI-MS can be used as a way to freeze protein oligomers in time, complications arise when a protein could simultaneously populate a number of states with the same mass-to-charge ratio [97]. This complication makes it difficult to quantify different size oligomers that have the same mass-to-charge ratio.

### 3.4. Ion Mobility (IM)-MS

IM-MS is capable of separating ions by both their shape and charge, which has rendered it a successful technique for the separation of conformers of various shapes arising from a single protein [94,99–101]. Ions are separated in time according to their cross sections by passing them through a drift cell containing helium gas under the influence of a weak electric field [102]. The flight times are combined with the drift times to yield the mass-to-charge IM distributions for all ions in the sample. The ability of IM-MS to separate species that differ in shape or size but have the same mass-to-charge ratio has made this technique a powerful tool for analyses of the early stages of Aβ oligomerization.

**Figure 7:**
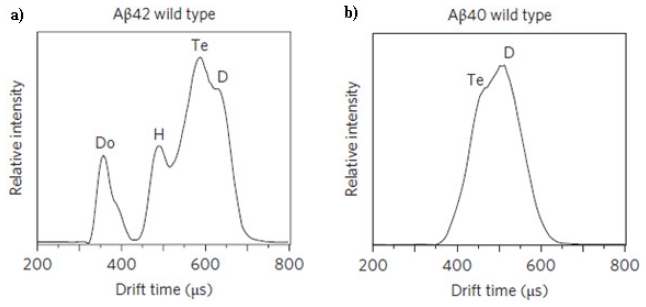
IM-MS arrival time distributions for (**a**) 30 μM Aβ_1-42_ in 49.5% H_2_O, 49.5% acetonitrile, and 1% NH_4_OH and (**b**) 30 μM Aβ_1-40_ in ammonium acetate (pH 7.4). *D* = dimer, Te = tetramer, *H* = hexamer, Do = dodecamer with a *z*/*n* = −5/2. Figure 7a adapted with permission from [103]. Copyright (2005) American Chemical Society. Figure 7b adapted by permission from Macmillan Publishers Ltd.: Nature Chemistry [104], copyright (2009).

Various research groups have utilized IM-MS to gain a better understanding of the early events of Aβ aggregation. Aβ_1-40_ conformational states in freshly dissolved and aggregated solutions have been studied by Iurascu *et al*. [48]. Two different conformational states were obtained for freshly dissolved Aβ_1-40_ and the soluble fraction obtained by Aβ_1-40_ incubation for 5 days at 37 °C and pH 7.5. Bernstein *et al*. used IM-MS to study the aggregation of Aβ_1-42_ *versus* Aβ_1-42_ with a Phe19→Pro19 substitution [103]. Monomers and large oligomers were produced by unfiltered Aβ_1-42_, while protein passed through a 10,000 amu filter yielded monomer, dimer, tetramer, hexamer, and an aggregate of two hexamers. In contrast, the Pro19 alloform produced monomer, dimer, trimer, and tetramer but no large oligomers. In a more recent study by Bernstein *et al*., a mechanism for Aβ_1-40_ and Aβ_1-42_ oligomerization and eventually fibril formation was postulated [104]. Using IM-MS, this group was able to determine the shape and size of Aβ_1-40_ and Aβ_1-42_ oligomers. Aβ_1-40_ oligomerization proceeded via the formation of dimer and tetramer followed by the very slow formation of fibrils containing a β-sheet structure. In contrast, Aβ_1-42_ proceeded via the formation of dimer, tetramer, and a hexameric paranucleus followed either by the formation of dodecameric species or the slow conversion into fibrils containing a β-sheet structure. Representative IM-MS data obtained by Berstein *et al*. for Aβ_1-42_ and Aβ_1-40_ are shown in Figure 7. Similar findings about the early oligomerization behavior of Aβ_1-40_ and Aβ_1-42_ were obtained by Murray *et al*. using IM-MS [102]. In addition, these researchers found that in an equimolar mixture of Aβ_1-40_ and Aβ_1-42_, Aβ_1-40_ inhibited the formation of higher molecular weight oligomers by Aβ_1-42_. This result suggests that Aβ_1-40_ could sequester Aβ_1-42_ into stable tetramers and prevent the further oligomerization of Aβ_1-42_ into dodecameric species.

### 3.5. Fluorescence Correlation Spectroscopy

Fluorescence correlation spectroscopy (FCS) has also been utilized to gain information about the size of Aβ species formed throughout aggregation [24,105–107]. FCS was originally developed by Eigen and Rigler in the early 1990s [108]. In FCS, unlabeled protein is combined with fluorescently labeled protein and, at various times throughout aggregation, the fluorescent dye is excited by a sharply focused laser beam. The emitted fluorescence of a small number of molecules in solution is observed. The fluorescence intensity fluctuates due to Brownian motion of the particles, and an intensity correlation function can be used to determine the average number and average diffusion time (*i.e.*, molecular size) of molecules. Advantages of FCS include high sensitivity (nM range and below), ability to examine a wide range of molecular sizes (*i.e.*, monomer, oligomer, fibrils) [109], fast analysis times [109], and small sample volumes (femtoliter) [110]. In addition, no pre-separation step is required for the determination of particle radius via FCS. However, assumptions must be made about the kinetics of the aggregation process as well as molecular shape in order to determine molecular weight.

**Figure 8:**
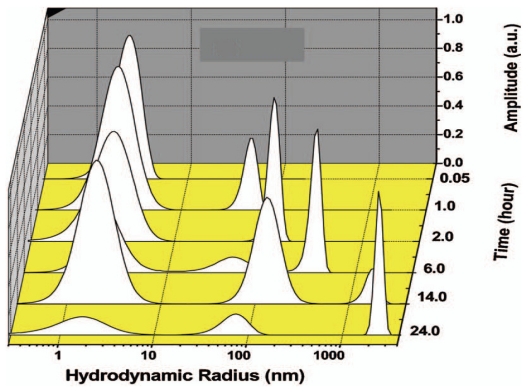
Size distributions obtained via FCS for Aβ_1-40_ dissolved in 2.8 mM NaOH, diluted to 10 μM in HEPES (pH 7.4), and incubated at room temperature. Sample taken at ~3 min shows predominantly monomeric species with the formation of intermediate aggregates of 20–100 nm after 1 h and further growth into larger aggregates >1000 nm after 24 h. Adapted with permission from [107]. Copyright (2008), American Institute of Physics.

Various researchers have employed FCS to monitor Aβ aggregation. A study by Matsumura *et al.* utilized FCS to monitor the aggregation of Aβ_1-40_ and Aβ_1-42_ and observed distinct aggregation pathways, dependent upon incubation conditions, that resulted in the formation of either oligomeric species or fibrils [24]. Two different site-specific labels at either the N-terminus or Lys^16^ were used to monitor aggregation. One pathway involved the formation of 10–15 nm spherical Aβ_1-42_ assemblies of ~330 kDa, termed amylospheroids (ASPDs), appearing after 5 h of gentle agitation of a 50 μM Aβ_1-42_ solution in F12 buffer at 4 °C. These ASPDs were formed from Aβ species of ~12.7 kDa initially present in solution. In addition, the aggregation pathways were similar for the N-terminus and Lys^16^ site-specific labels. An alternative pathway involved fibril formation from 100 μM Aβ_1-40_ solutions in Dulbecco’s PBS (pH 3.5) with gentle agitation at 4 °C. This pathway began with dimer formation at 0 h, followed by the formation of intermediate sized species of 15–40 nm after 2–9 h. Eventually, larger molecular weight fibrils (14,000 kDa) were formed after 24 h using Aβ labeled site-specifically at Lys^16^. However, much larger aggregates (120,000 and 3,900,000,000 kDa) were formed after 24 h using Aβ labeled site-specifically at the N-terminus. It was thus postulated that the Lys^16^ fluorescent probe interfered with aggregation into larger fibrils. By employing oligomer formation conditions, Cizas *et al.* used FCS to observe much smaller Aβ_1-42_ oligomers [105]. They dissolved Aβ_1-42_ in HFIP with subsequent dilution into de-ionized water and incubation at 20 °C with or without agitation (500 rpm) for 24 h. The average radius observed for unagitated samples was ~3.4 nm while the radius for agitated samples was ~8 nm. Garai *et al.* have applied FCS to monitor the Aβ_1-40_ aggregation process when monomer is initially the predominant species present in solution (Figure 8, time = 0.05 h) [107]. After 1 h, intermediate aggregates of 20–100 nm formed and grew to sizes >1000 nm after 24 h (Figure 8, time = 2–24 h). These Aβ_1-40_ intermediate sizes are larger than those observed by Mastmura *et al.* and could be due to different sample preparations or the presence of different Aβ species at 0 h. These studies again show that although Aβ_1-40_ and Aβ_1-42_ differ by only two amino acid residues, the aggregates formed are considerably different.

### 3.6. Summary of Spectroscopic Methods

MS is capable of detecting low oligomer concentrations but is expensive and has difficulty separating species with identical mass-to-charge ratios such as Aβ aggregates [96,97]. To address this problem, MS is often coupled with an upstream separation technique such as SDS-PAGE [48,95]. In addition, IM-MS has been utilized for the separation of different sizes and conformations of Aβ_1-40_ and Aβ_1-42_ with promising results for small oligomers. However, the addition of a step such as IM also increases the time needed for analysis and therefore decreases the chances of detecting transient species. Consistent results for Aβ_1-40_ and Aβ_1-42_ oligomer sizes formed during the earliest events of aggregation have been obtained using MS techniques. However, the detection of larger Aβ_1-40_ and Aβ_1-42_ oligomeric species ranging from ~32–~100 kDa has not been achieved using MS. FCS does not require a pre-separation step to determine the particle radius of species in a sample. This technique has been successfully applied for the detection of small and intermediate sized Aβ oligomers as well as large Aβ fibrils. However, FCS yields average values of particle radius for a population of aggregates and not individual particle sizes or their distributions.

## 4. Additional Techniques Utilized for Aβ Aggregate Size Determinations

### 4.1. Light Scattering Techniques

Light scattering techniques have been used to measure Aβ aggregate sizes. Classical, or multi-angle, light scattering (MALS) employs a well collimated, single frequency light beam to illuminate a sample of macromolecules [111]. When incident light interacts with the macromolecules in solution, an oscillating dipole is induced and the light is re-radiated, or scattered [112]. Aggregated structures induce coherent scattering, and as a result the intensity of scattered light is dependent upon molar mass. Furthermore, destructive and constructive scattering that result from the independent scattering of individual molecular elements can give rise to an angular dependence of the scattered light, which is a function of the size of the molecule. Thus, the intensity of the scattered light is measured as a function of scattering angle, often referred to as Rayleigh scattering, to yield the molar mass and root mean square (rms) radius of the macromolecules [112]. MALS is ideal for characterizing larger assemblies (>10 nm). In contrast, for analyses in which smaller molecules are present in solution, dynamic light scattering (DLS), also known as quasi-elastic light scattering (QELS), is used. DLS employs a fast photon counter to measure time dependent fluctuations in scattered light at a single angle (usually 90°), which are related to the rate of diffusion of the macromolecules [112,113]. Measurement of diffusion rates allows calculation of the hydrodynamic radius (*R*_H_) of macromolecules using the Stokes-Einstein equation [113]. When used as standalone techniques, MALS yields the weight-averaged molar mass for all molecules in solution. While DLS can distinguish populations that differ in size by a factor of five or more, individual peaks exhibit a high degree of polydispersity. Therefore, it is often necessary to utilize a pre-separation step in conjunction with light scattering to obtain an accurate estimate of the relative amounts of individual aggregates present in solution. In addition, the exponential dependence of scattering on aggregate size prohibits the detection of low quantities of small aggregates in the presence of larger species.

**Figure 9:**
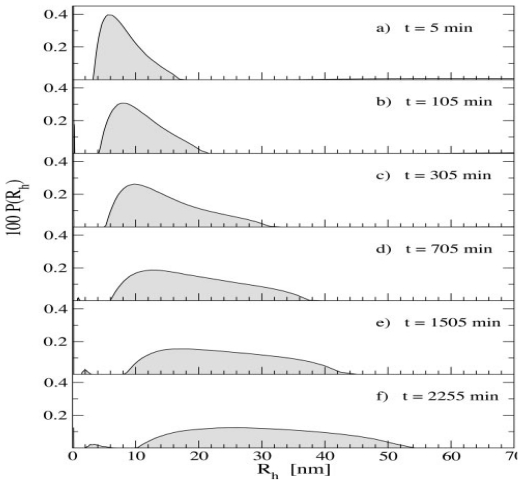
Time evolution of *R*_H_ for a 185 μM Aβ_1-40_ sample incubated at pH 3.1 and 37 °C. Distributions were determined using a constrained regularization method. Reprinted with permission from [117]. Copyright (2005) The American Society for Biochemistry and Molecular Biology.

Various researchers have utilized MALS and/or DLS to characterize Aβ assemblies formed throughout aggregation [114–117]. Carrotta *et al.* utilized both MALS and DLS to monitor the aggregation of a 185 μM Aβ_1-40_ sample at pH 3.1 and 37 °C [117]. DLS was used to characterize aggregate sizes formed during the early stages of aggregation up to ~38 h, as shown in Figure 9. After 5 min (Figure 9a), an average *R*_H_ of 7 nm was obtained. The size distribution became more polydisperse over time and ranged from 10–52 nm after 37 h (Figure 9f). However, only average size distributions could be obtained and no information was reported about the concentrations of each aggregate species (*i.e.*, monomer, dimer, *etc*.). Larger aggregates (hundreds of microns) were formed after 2 weeks as detected by MALS.

Similar to these findings, Lomakin *et al.* observed using DLS the initial formation of a spherocylindrical micelle with average *R*_H_ of 7 nm immediately following dissolution of Aβ_1-40_ at pH 2 [115]. In addition, they reported two different kinetic patterns for aggregation of Aβ_1-40_ prepared at a concentration either above or below the critical micelle concentration (CMC) of 100 μM [114] Complimentary DLS and MALS studies by Murphy and Pallitto also demonstrated an effect of Aβ concentration upon aggregate formation [118]. They demonstrated that dilution of Aβ_1-40_ from urea into PBS yielded larger aggregates at lower protein concentrations, while the increase in *R*_H_ for aggregates was proportional to the protein concentration. In addition, MALS data indicated that the linear density of aggregates increased with protein concentration. Thunecke *et al.* have utilized MALS and DLS to study the aggregation of Aβ_1-40_ and Aβ_1-42_ in acetonitrile-water mixtures [116]. At the onset of aggregation, Aβ_1-42_ was present as a 2 nm oligomer and rapidly formed fibrils with a length <50 nm within 4.5 h. In contrast, Aβ_1-40_ initially exhibited large aggregates that grew 70 times slower than aggregates of Aβ_1-42_. However, the presence of these large aggregates may preclude observation of a separate population of oligomers. These findings highlight differences in the dissolution and aggregation of Aβ_1-40_ and Aβ_1-42_.

### 4.2. Light Scattering in Combination with Other Techniques

Because light scattering techniques provide information about the weight-average molar mass and radius for all molecules in solution, they are often coupled with a pre-separation technique such as asymmetric field flow fractionation (AFFF) [119] or SEC [25,120,121] to better characterize individual Aβ oligomeric species. A study by Nichols *et al.* utilized MALS with SEC as well as DLS to characterize Aβ_1-40_ protofibrils following growth by monomer elongation or lateral association [120]. They found that protofibrils isolated by SEC exhibited an average *R*_H_ of 51 nm and molecular weight, determined via MALS of 30,000 kDa. Protofibrils that had grown by monomer deposition had an average *R*_H_ of 143 nm and molecular weight of 57,000 kDa, while protofibrils that had grown by lateral association had an average *R*_H_ of 104 nm and molecular weight of 86,000 kDa. Furthermore, SEC-MALS revealed that the mass per unit length of protofibrils was unchanged during elongation, but was increased following association. The temporal change in size of Aβ_1-40_ protofibrils isolated by SEC has also been monitored via DLS by Walsh *et al.* [121]. The initial average *R*_H_ for protofibrils isolated by SEC was ~27.8 nm, and protofibril size grew to 80.6 nm over a period of 9 days when 17 μM Aβ_1-40_ in Tris-HCl (pH 7.4) containing 0.04% w/v sodium azide was incubated at room temperature.

AFFF is another technique that has been coupled with light scattering to estimate the molecular weight of individual Aβ aggregates. AFFF exploits the parabolic flow profile created by the laminar flow of a sample through a thin, parallel plate flow channel, where the lower surface is solvent permeable [122]. A perpendicular force applied to the laminar flow stream drives molecules towards the permeable boundary layer of the channel [123]. Because Brownian motion of the particles creates a counteracting force, smaller particles localize higher in the channel leading to separation of different molecular sizes, with smaller molecules eluting first [122]. Rambaldi *et al.* utilized AFFF-MALS to monitor the aggregation of Aβ_1-42_ in PBS (pH 7.4) at room temperature over 24 h [119]. At 0 h, two major peaks were obtained corresponding to molecular weights of ~60 kDa and ~1000–100,000 kDa. In addition, the retention time of the ~60 kDa species decreased between 0 and 4 h, corresponding to an increase in aggregate size of 6.5–4.7 nm. The intensity of the two peaks also decreased over 24 h, possibly due to irreversible adsorption of the sample to the permeable surface. Although AFFF-MALS has several advantages, including gentle, rapid, and non-destructive separation, improvements to the ultrafiltration membrane are critical to enhance analysis capabilities. In addition, the smallest molecular weight cutoff for membranes is 5 kDa, making detection of Aβ monomeric species difficult.

### 4.3. Centrifugation

Centrifugation has also been explored as a method for determining Aβ size. Here, sedimentation coefficient (s) values can be correlated with molecular weight. Mok and Howlett provide a nice overview of sedimentation velocity centrifugation in the context of Aβ analysis [124]. Ward *et al.* used density gradient centrifugation to fractionate Aβ_1-40_ samples incubated at pH 7.4, 35 °C for 30 min, 18 h, or 18 days [26]. Using SDS-PAGE with Western blotting to analyze sedimented samples, they found that Aβ_1-40_ incubated for 18 h contained only small molecular weight oligomers (4–17 kDa), while Aβ_1-40_ incubated for 18 days showed the presence of a >250 kDa band as well as significant streaking, indicating other unresolved sizes. Huang *et al.* used analytical ultracentrifugation to compare Aβ_1-40_ samples prepared at pH 3, 5, and 7 [125]. They determined that at pH 5 there were no soluble aggregate species. At pH 7, they identified small oligomers with an average molar mass of 12.1 kDa, and at pH 3 they identified a range of aggregate sizes with an average molecular weight of 1 MDa. Nagel-Stefer *et al.* also used sedimentation velocity centrifugation for the analysis of Aβ_1-42_ samples after 5 days of agitation at room temperature and were able to detect “globular species” ranging in size from ~270 kDa–3.8 MDa as well as even larger aggregates [126]. Interestingly, they also compared three different simulation methods for determining molecular weight from sedimentation values and obtained molar masses that differed by approximately one order of magnitude.

### 4.4. Size Exclusion Chromatography

SEC, a chromatographic technique, separates molecules based on molecular hydrodynamic volume or size. Molecules too large to penetrate the pores of the column packing material elute in the void volume, while smaller molecules travel through the pores and elute at later times. Globular proteins are often used as standards to estimate the size of Aβ oligomers. However, since Aβ is a linear, hydrophobic peptide, comparisons between the elution behavior of Aβ oligomers and size standards are difficult [127]. In addition, the sample is subjected to a several-fold dilution, which facilitates the dissociation of small unstable oligomers [128], thereby precluding the detection and size estimation of these species.

**Figure 10:**
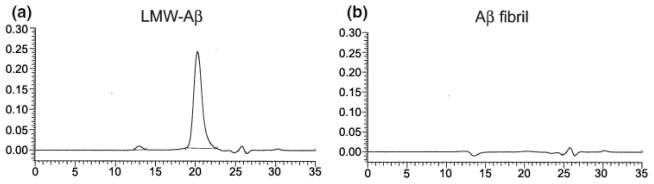

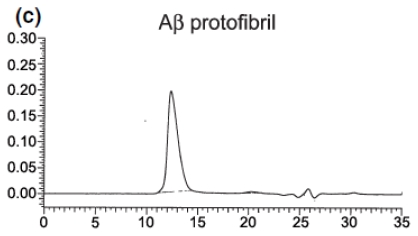
HPLC-SEC chromatograms of Aβ aggregates produced using sample preparations of 50 μM synthetic Aβ designed to optimize (**a**) low molecular weight Aβ_1-40_ oligomers and (**c**) Aβ_1-42_ protofibrils. To ensure that insoluble fibrils were not present in solution, these species were removed via centrifugation prior to analysis, and this was confirmed by an absence of SEC signal in (**b**), a fibrillar Aβ_1-42_ preparation. Absorbance at 214 nm is given on the y-axis and retention time is given on the x-axis. Adapted from [130] published by John Wiley and Sons, © 2007 The Authors Journal Compilation © 2007 International Society for Neurochemistry.

Although SEC is typically utilized in conjunction with another technique, SEC as a standalone technique has been employed for the study of Aβ aggregates [129–131]. Englund *et al.* used SEC to detect low molecular weight Aβ aggregates, Aβ protofibrils, and Aβ fibrils formed using different Aβ sample preparations [130]. The size of low molecular weight Aβ aggregates ranged from 4–20 kDa (Figure 10, panel a), while Aβ protofibrils were >100 kDa (Figure 10, panel c). A more narrow size distribution of Aβ_1-42_ oligomers of 24 ± 3 kDa (pentamer–hexamer) has been obtained by Ahmed *et al.* with SEC [129]. This resolution was achieved by stabilizing Aβ_1-42_ oligomers at a low temperature (4 °C) and low salt concentration (10 mM NaCl). Zheng *et al.* have analyzed via SEC freshly prepared 1 mg/mL Aβ_1-40_ in PBS (pH 7.4), diluted from DMSO, and achieved resolution of an Aβ_1-40_ trimer with molecular weight of 11.6–15.7 kDa [131]. The difference in sizes obtained by Ahmed *et al.* and Zheng *et al.* most likely result from differences in sample preparation. While these studies show promising results for resolution of a single low molecular weight Aβ oligomer, the resolution of individual intermediate Aβ oligomeric sizes formed during aggregation has not been achieved using SEC as a standalone technique.

### 4.5. Summary of Additional Aβ Aggregate Size Determination Techniques

Light scattering techniques, such as MALS and DLS, have been used to detect both small and large Aβ aggregates. DLS is more suitable for the detection of smaller aggregates and gives information about aggregate size, or *R*_H_, while MALS has been utilized for the detection of larger Aβ species, including fibrils, and can provide information about molar mass. MALS and DLS, however, give a weight-average molar mass or *R*_H_ for all molecules in solution and must be coupled to another technique in order to increase the resolution of individual sizes. SEC as a standalone technique has been utilized to detect low molecular weight Aβ oligomers and protofibrils, and SEC-MALS has been used to characterize protofibrils formed via different growth mechanisms. However, due to the dilutions required by SEC, small unstable oligomers are often dissociated, thereby precluding their analysis. AFFF-MALS does not require a pre-fractionation step and has been used to separate Aβ oligomers of ~60 kDa from larger species. This technique yields a gentle, non-destructive separation of molecules. However, further improvements to the ultrafiltration membranes must be made in order to reduce adsorption of the sample to the membrane. Centrifugation has also been explored for the separation of small oligomers (4–17 kDa) and larger species (>250 kDa) but requires an uncertain correlation of sedimentation coefficients with molar mass. Each of these techniques are suitable for the detection of a wide range of Aβ aggregates present throughout aggregation but present difficulties with respect to the resolution and quantification of individual Aβ aggregate sizes.

## 5. Techniques Utilized for Aβ Oligomer Identification

While this review focuses primarily on techniques capable of qualitatively determining the size of Aβ oligomers, techniques that can identify the presence of oligomers, without providing information about oligomer size, are also available. Although qualitative in nature, we have chosen to briefly discuss two of these techniques, dot blot and ELISA, as a result of their frequent use and emerging interest.

### 5.1. Dot Blot

**Figure 11:**
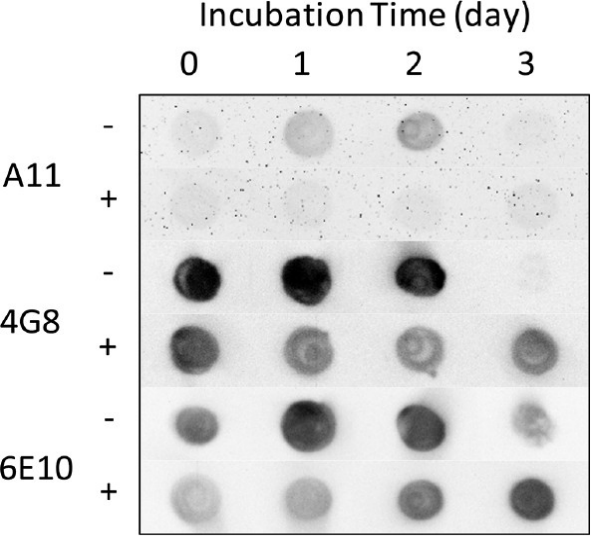
Aβ aggregation monitored via dot blot. A 50 μM Aβ_1-40_ sample was incubated in PBS (pH 7.4) at 37 °C in the presence (+) or absence (−) 3 x Brilliant Blue G (BBG) inhibitor. Samples were taken on the indicated days and spotted on a nitrocellulose membrane. Oligomer-specific A11 antibody and Aβ-sequence specific antibodies 4G8 and 6E10 were used to detect aggregates. Reprinted with permission from [57]. Copyright (2011) American Chemical Society.

Dot blots employ a protein captured upon a membrane as a spot, or dot. A primary antibody binds to the protein epitope of interest followed by the binding of a secondary antibody to facilitate detection. When dot blots are probed with antibodies that specifically recognize oligomeric Aβ, they can confirm the presence of oligomers but give no information about aggregate size. Three different Aβ antibodies, oligomer-specific A11 or sequence specific 4G8 and 6E10 (see Table 1 for Aβ binding epitopes), were employed in conjunction with a dot blot assay for detection of aggregating Aβ by Wong *et al.* [57]. Aβ_1-40_ was diluted to 50 μM in PBS (pH 7.4) and incubated at 37 °C. At times ranging from 0–3 days, a sample was analyzed via dot blot, as shown in Figure 11. A11 binding revealed the transient appearance of oligomers in uninhibited samples, while detection via 4G8 and 6E10 remained constant until later times when signals decreased, presumably due to masking of binding sites following aggregation. Changes in these patterns in the presence of inhibitor demonstrated the ability of the inhibitor to prevent oligomer formation and slow the evolution of larger aggregates. Necula *et al.* used a dot blot assay to monitor the oligomerization of Aβ_1-42_ dissolved in 100 mM NaOH, diluted to 45 μM in PBS (pH 7.4), and incubated at room temperature for 10 days [132]. Similar to Wong *et al.*, they probed the specificity of three different antibodies, oligomer-specific A11 and sequence specific 6E10 and 4G8. At 0 days, 6E10 and 4G8 strongly reacted with Aβ_1-42_ aliquots, while A11 reacted weakly, indicating that only monomeric species were present. A strong immunoreactivity for A11 was observed after 4 days and continued to increase in intensity over 10 days, similar to results obtained by Wong *et al*. Again, this was accompanied by a decrease in immunoreactivity of 6E10 and 4G8.

### 5.2. Enzyme-Linked Immunosorbent Assay

ELISA is a commonly used technique for the identification of Aβ oligomers. ELISA may be used in a traditional or sandwich assay format. In the traditional format, protein adsorbed at a surface can be detected using a primary antibody that is specific for Aβ oligomers (see Table 1). This primary antibody can be directly linked to an enzyme that converts added substrate to a detectable signal (direct ELISA) or can be coupled with a secondary antibody containing the enzyme moiety (indirect ELISA). The latter format serves to enhance the assay signal. Alternatively, in the sandwich ELISA format, a sequence-specific capture antibody (see Table 1) adsorbed onto the surface is used to capture Aβ protein, which is subsequently detected using the same sequence-specific antibody, such that only Aβ species containing multiple monomeric units, and therefore multiple epitopes, are detected [130,133]. Consequently, this sandwich ELISA will recognize only aggregated Aβ, but not Aβ monomer. Although ELISA can identify the presence of Aβ oligomers in a sample, this technique is not capable of determining sizes of these oligomeric species. Therefore, ELISA is most advantageous for the detection of oligomeric Aβ within a sample containing many different proteins.

Various researchers have utilized ELISA for the detection of Aβ oligomers [128,130,133–135]. A study by Englund *et al.* employed a sandwich ELISA with monoclonal antibody 158 for the detection of low molecular weight oligomeric Aβ_1-40_ produced by dissolving Aβ_1-40_ in 10 mM NaOH with dilution to 50 μM in 2 X PBS and Aβ_1-42_ protofibrils produced by dissolving Aβ_1-42_ in 10 mM NaOH with dilution to 443 μM in 2 X PBS and incubation overnight at 37 °C [130]. Gonzales *et al.* utilized a similar ELISA assay to detect low molecular weight Aβ_1-42_ formed by dissolving Aβ_1-42_ in HFIP with dilution to 200 nM in PBS (pH 7.2) and incubation at 37 °C for 24 h [133]. The size of these species was confirmed with PAGE to be tetramer and pentamer; however, the bands were very faint, indicating the superior sensitivity of the ELISA assay for these oligomeric species. A detection limit for Aβ_1-40_ oligomers of 80 nM was obtained in these studies.

### 5.3. Summary of Aβ Oligomer Identification Techniques

Dot blots and ELISAs have been employed to detect oligomeric Aβ assemblies. Dot blots have been used to observe the transient evolution of oligomers during aggregation, but provided no information about Aβ aggregate size. Low molecular weight Aβ oligomers and Aβ protofibrils have been detected via ELISA at nanomolar concentrations. However, PAGE was required to estimate the size of these species. Thus, these techniques can sensitively confirm the presence of oligomers but yield no size information.

## 6. Conclusions

This review describes a variety of techniques, summarized in Table 2, that are currently utilized to determine the size or presence of Aβ aggregates, with a focus upon oligomeric species. These techniques have been explored for the quantitative detection of different aggregate sizes with various limitations to their resolution, dependence on pre-analysis procedures, sensitivity, cost, *etc*. Electrophoretic techniques, such as SDS-PAGE, Western blotting, and CE, are widely used for size-based separations of Aβ aggregates. In particular, SDS-PAGE and Western blotting are suitable for the detection of monomeric and small oligomeric Aβ species. The separation of larger oligomers via SDS-PAGE is more difficult due to the sensitivity of these sizes to denaturing conditions, which can result in aggregate decomposition during analysis. The recent development of antibodies specific for Aβ oligomers has led to an increase in the application of Western blotting, dot blotting, and ELISA to study Aβ aggregation. However, the detection limits of Western and dot blotting prohibit study of physiologically relevant Aβ concentrations. While more sensitive, ELISA is better suited for the identification of specific analytes, such as Aβ oligomers, present within a mixed population but cannot distinguish individual oligomer sizes. CE with LIF detection offers a highly sensitive detection of physiologically relevant concentrations, but the application of CE to amyloid aggregation analyses is still in the early stages. MS is another commonly used technique for Aβ aggregate size-based separations. MS has been successfully used to detect small oligomeric species (especially IM-MS) but quantitative analyses of aggregate size may be limited by the pre-separation step, the ability to differentiate species with highly similar charge-to-mass ratios, and high equipment costs. FCS, MALS, and DLS may be utilized for determination of Aβ aggregate size, but yield a weight-averaged molecular weight of species, thereby limiting the resolution of individual Aβ aggregate species. Centrifugation has been used to examine small oligomeric species up to large fibrils; however, selection of the method for determination of molar mass from sedimentation coefficients can play an important role in size estimation. SEC may be coupled with these approaches or used as a standalone technique; however, SEC is complicated by dilution of the analyte during separation, inadequate resolution of intermediate oligomeric species, and limited utility of size standards.

Although each of the methods discussed in this review has the capability to determine Aβ aggregate size, the pathogenic events that initiate the misfolding of Aβ and formation of aggregate species remain elusive. Hence, there is a continued need for improvement of these techniques in order to realize the effective detection of small size differences in Aβ oligomers. In order to leverage the advantages of each Aβ detection method, a combination of approaches must be utilized, allowing validation of findings from different techniques and a better understanding of the early events of the Aβ aggregation process.

## Figures and Tables

**Table 1:** Antibodies used for amyloid-β protein (Aβ) detection in Western blot analysis and their respective Aβ recognition motifs.

Antibody	Recognition Motif	Monoclonal/Polyclonal	References
6E10	Aβ_1-17_	Monoclonal	[50–53]
Ab9	Aβ_1-16_	Monoclonal	[54]
6C6	Aβ_1-16_	Monoclonal	[50]
4G8	Aβ_17-24_	Monoclonal	[50]
2G3	Aβ_31-40_	Monoclonal	[55]
BA-27	Aβ_1-40_, C-terminal	Monoclonal	[56]
BC-05	Aβ_1-42_, C-terminal	Monoclonal	[56]
A8	amyloid oligomers	Monoclonal	[45]
A11	amyloid oligomers	Monoclonal	[10,57,58]
NU-4	amyloid oligomers	Monoclonal	[59]
OC	amyloid fibrils	Polycolonal	[60]

**Table 2:** Summary of techniques for the quantitative detection and/or identification of Aβ aggregate sizes formed throughout the aggregation process.

Technique	Advantages	Disadvantages	Aggregate Sizes Detected	References
SDS-PAGE	SDS offers strong sizebased separation	SDS may induce non-native behavior and destabilize oligomers Gel smearing	4.5–20 kDa, >83 kDa	[45,46]
Native PAGE	Ability to separate based on charge and hydrodynamic size	Gel smearing	8–20 kDa, high molecular weight	[48,49]
Western Blotting	High sensitivity and specificity,	Requires specific and expensive antibodies Incomplete transfer of proteins onto membrane Technically demanding	4–16 kDa, 16.5–25 kDa, 30–97 kDa (with SDS-PAGE)	[45,50–53,55]
Capillary and Microfluidic Capillary Electrophoresis	Fast, highly sensitive separation of proteins based on charge and hydrodynamic size Low sample volume	Low resolution of intermediate sized Aβ oligomers Irreproducibility	4–50 kDa, >50 kDa, fibrils	[72–74]
Mass Spectrometry	Fast data acquisition Can identify multiple species with different mass-to-charge ratios	Inability to distinguish molecules with overlapping mass-to-charge ratios (MALDI, ESI) Expensive Labor intensive	4–24 kDa, ~48 kDa, fibrils,	[48,95,98, 102–104]
Fluorescence Correlation Spectroscopy	High sensitivity, ability to look at wide range of sizes within a sample Fast analysis time Low sample volume	Relies on assumptions about shape and kinetics of protein to determine molecular weight Yields average molecular weight values	~10 nm–1 μm (small oligomers– aggregates)	[24,105,107]
Light Scattering	Direct measurement of molar mass and radius (MALS) Simultaneous detection of multiple populations within a sample (DLS)	Yields weight-average molar mass and not size of individual species or their distribution Exponential dependence of scattering on aggregate size	>10 kDa (MALS) 1 nm–1 μm (DLS)	[112,116,117]
Centrifugation	Ability to detect a wide range of sizes (oligomers–fibrils) Fast analysis time	Theoretical size estimate depends on appropriate assumptions in the model	4–17 kDa, >250 kDa, ~270 kDa–3.8 MDa	[26,126]
Size Exclusion Chromatography	Well established technique	Leads to sample dilution which can dissociate unstable oligomers Comparisons between elution behavior of oligomers and globular protein standards make molecular weight estimations difficult	4–20 kDa, 24 kDa, >100 kDa	[129–131]
Enzyme-Linked Immunosorbent Assay	Highly sensitive and specific Ability to measure specific analytes within a crude preparation Versatile	Gives information about presence of oligomers and not size Requires expensive and specific antibodies	No size determination	[130,133]
Dot Blot	Straight-forward, rapid technique	Gives information about presence of oligomers and not size Requires expensive and specific antibodies	No size determination	[57,132]
